# Development of a permanent vacuum hollow prism air refractometer for use in dimensional metrology

**DOI:** 10.1038/s41598-021-88697-4

**Published:** 2021-04-29

**Authors:** O. Kruger, N. Chetty

**Affiliations:** 1 School of Chemistry and Physics, Discipline of Physics, University of KwaZulu-Natal (PMB), Private Bag X01, Scottsville, 3209 South Africa; 2 National Metrology Institute of South Africa, CSIR Campus, Private Bag X34, Pretoria, 0001 South Africa

**Keywords:** Engineering, Physics

## Abstract

Refractive index measurements are required when light is used as the basis of a measurement system. In dimensional metrology, refractive index measurements are used to compensate for the change in the speed of light. This is crucial because the SI unit for the metre is defined as the speed of light in a vacuum. Air refractometers are the most accurate way to measure the speed of light in air. Many research works to date have been performed to measure the refractive index of air using refractometers. This research uses a commercial prism as the vacuum etalon instead of the tube that is used most often. This novelty and newness of our research were to focus on the design, fabrication and testing of a refractometer which uses a permanent vacuum for ease of use but that will still have the same accuracy of other refractometers currently in use. Modifications to existing designs improved the long-term stability compared to other prism refractometers and are also potentially more accurate than tube refractometers. The results achieved with this permanent vacuum refractometer are accurate to 8.4 × 10^–8^, which compares favourably with other refractometers on accuracy. It also has the added advantage that it does not require a vacuum pump, and with added laser path improved long term stability but still portable and robust enough to use in everyday applications.

## Introduction

Refractive index measurements in the medical industry are used to measure, for example, the specific gravity of human urine to determine kidney function^1–3^. In veterinary medicine, it is used to measure the plasma protein and in-cell measurements in biological tissues^4–6^. It is also used in the design of optical systems, especially for the medical industry in eye difference design^7^. Refractive index measurements are also used to determine the sugar content in various beverages, including wine^8^. The application and impact of refractive index measurements are well captured by Singh^9^.

This paper focuses on refractive index measurements in metrology and more especially dimensional metrology, an area that requires highly accurate measurements. Most measurements are performed at the highest sensitivity for accurate results by utilising laser light to perform the distance measurements. These measurements require the refractive index to be known to a high degree of accuracy.

Since 1983, the metre has been defined as the length of the path travelled by light in a vacuum in 1/299 792 458 of a second^10–16^. However, most dimensional and length measurements are performed in the air. The refractive index in air changes with environmental conditions (air pressure, air temperature, humidity and CO_2_), and thus, the refractive index of air at the specific conditions must be calculated to make corrections compared to the definition of the metre. This is exacting work and sometimes difficult to achieve practically in the environment.

To calculate the refractive index, the speed (velocity) of light in a vacuum, *c*, is divided by *v′*, the velocity of light in air at the time the measurements are taken.

The refractive index is thus:

1 $$ n = \frac{c}{{v^{\prime}}}, $$

where *n* is the refractive index, *c* is the velocity of light in a vacuum, and *v′* is the velocity of light in a particular medium, in this case, air.

Many research works to date have been performed to measure the refractive index of air using refractometers. This novelty and newness of our research were to focus on the design, fabrication and testing of a refractometer which uses a permanent vacuum for ease of use but that will still have the same accuracy of other refractometers currently in use. Our device would be suitable for use in everyday applications due to its simplicity of design and ease of use.

### Refractive index measurements and refractometers in dimensional measurements

The most common method to determine the refractive index is to measure the environmental conditions (air temperature, air pressure, relative humidity and CO_2_) and then calculate it by using equations from Edlin^17^, Birch^18^ or Ciddor^19^. Much research has been done on the differences between these equations by Bonsch and Potulski^20^ and Kruger and Chetty^21^. The original calculations were updated specifically for the moisture content of the air. Metrologists in dimensional laboratories and commercial manufacturers of laser measurement systems, such as Rensihaw^22^, Agilent^23^ and Zygo^24^, now mainly use the modified equation. Hence, it was decided to use the modified Edlin equation in this research.

Equation (2) is the modified Edlin equation used to calculate the refractive index of air^18^.

2 $$ \left( {n - 1} \right)_{Tp} = \frac{{p\left( {n - 1} \right)_{s} }}{93 214.60}.\frac{{1 + 10^{ - 8} p\left( {0.5953 - 0.009876T} \right)}}{{\left( {1 + 0.0036610T} \right)}}. $$

Here (*n* – 1) is the refractivity of standard air, the temperature T is expressed in degrees Celsius, the air pressure *p* in torr, standard air at 1 atmosphere and 15 °C.

The more accurate method to determine the refractive index is to use a refractometer that measures the refractive index directly by comparing an optical path in the air to the same optical path in a vacuum, as described by Bonsch and Potulski^20^ and Kruger and Chetty^21^. Research by Zhang et al.^25^ and Wu and Takahashi^26^ used frequency combs as the measuring system, and although these gave a very high resolution, the overall accuracy is similar to other designs due to the optical layout. The fact that the distance between the mirrors is not absolutely stable is also of concern.

The main reason the refractometer method is more accurate is that the weather station data have more technical difficulties^25–30^. Kruger and Chetty^21,31^ studied these difficulties and decided to focus the research on refractometers that will be used for highly accurate refractive index measurements. Kruger and Chetty^21,31^ developed a low-cost robust refractometer. Their research included the development of refractive index measurements and a robust refractometer that uses a commercial laser displacement interferometer together with a vacuum cylinder to measure the refractive index to parts in the 10^–8^.

This refractometer is very accurate and can be used daily because of its simplistic design, however, it requires a vacuum pump to perform the refractive index measurements. The vacuum pump must be close to the refractometer to achieve a good vacuum, which leads to the heat from the pump affecting the air temperature of the laser measurements. Another limitation to this refractometer is that repeatedly drawing a vacuum can lead to dust particles entering the vacuum cylinder, and these can affect the measurements through diffraction of the laser beam.

A further challenge with the traditional tube refractometer is the change in the optical thickness of the side windows in the tube refractometer. Bonsch and Potulski^20^ measured this to be 8 nm, and although it is very small, it adds to the inaccuracy of these tube refractometers.

These problems led to this research, the aim of which was to design, build and test a simple air refractometer that can be used in everyday laser-based measurements without the use of a vacuum pump and to eliminate the errors in tube refractometers as describe by Bonsch and Potulski^20^.

## Experimental details

Investigating a new refractometer led to using a hollow prism as the vacuum etalon. The concept was that the prism will have a permanent vacuum, consequently eliminating the use of a vacuum pump during measurements, thereby producing more reliable measurements.

**Figure 1:**
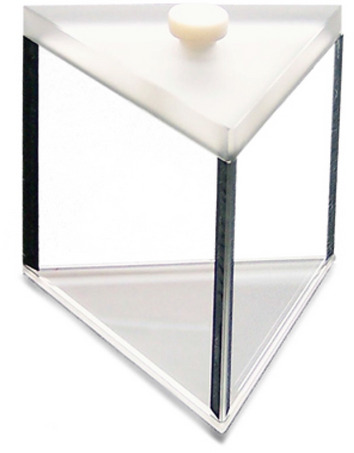
Hollow prism from 3B Scientific.

The prism used is a commercial prism that is normally used for the refractive index measurements of liquids^32^. This specific prism is from 3B Scientific and shown in Fig. 1^33^. Only basic specifications for the prism are available, and there is no specification for the optical flatness and parallelism of the side windows, which is critical and will be discussed in more detail later. The prism was modified by attaching a vacuum pipeline to the opening of the prism so that a vacuum could be drawn inside the prism.

An optical layout similar to the design of the previous cylinder refractometer by Kruger and Chetty^21^ was used. In this research, the vacuum cylinder was replaced with the above-mentioned prism. Instead of using a vacuum pump to generate the vacuum required to compare the air to vacuum ratio, the prism was moved across the laser beam to replace the optical path length from air to vacuum. This has the advantage of not requiring a vacuum pump during the measurements.

**Figure 2:**
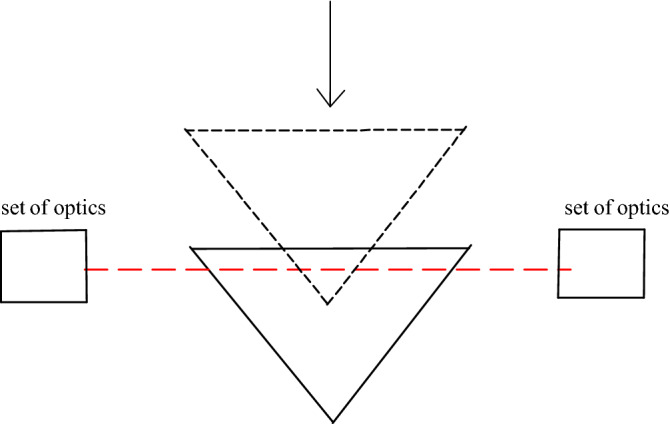
Schematic diagram of, with top view prism refractometer using laser displacement interferometer and translation stage.

Figure 2 shows a schematic layout of the prism refractometer. Any laser system could theoretically be used, but in this design, a commercial heterodyne laser (Zygo ZMI 2000 laser interferometer) was used. The frequency difference of the two laser beams is about 20 MHz^24^. Agilent angle interferometer optics (10770A) was used to split the two laser beams in the polarising beam splitter. It separates the two beams, f1 and f2, and directs them parallel and 32.62 mm apart^23^.

The vacuum prism was positioned in such a way that the one frequency of the laser, f2, passed through the prism, which is under vacuum and the other frequency, f1, passed above the prism in normal air. With the use of a translation stage, the prism was moved from position 1, the dotted prism, through laser beam f2 to position 2, the solid line prism, thereby replacing the air with a vacuum column.

**Figure 3:**
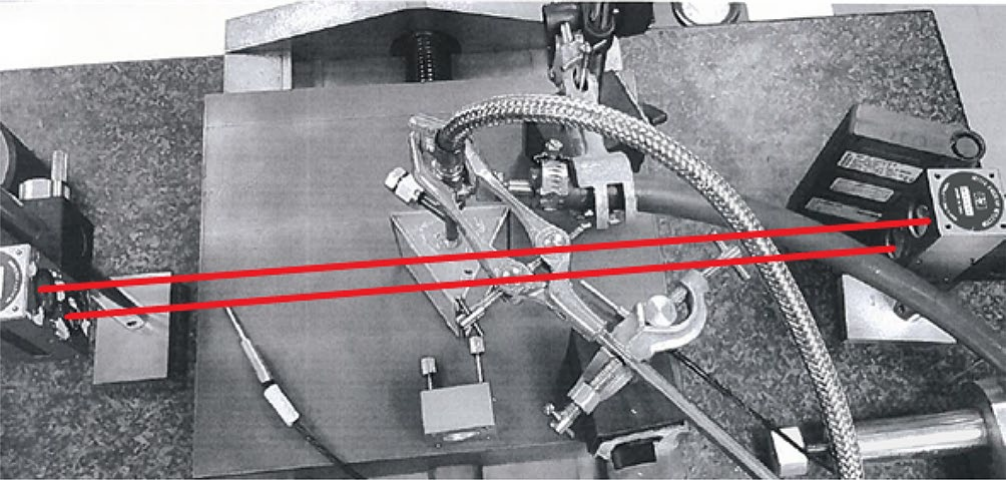
Prism refractometer with laser beams.

Figure 3 shows the prism placed on the translation stage where a displacement laser (HP/Agilent Model 5519A) was used to measure the displacement accurately.

The distance the translation stage is moved, $Lt$, together with the half-angle θ are used to calculate the light path in the vacuum inside the prism, *L.* The light path in a vacuum, *L*, is a relative change as the zero position for *L* is already in a small part of the vacuum.

3 $$ L = 2 \times \left( {tan\theta \times Lt} \right). $$

This method has also been researched by Renkens and Schellekens^34^, and there was a US patent filed in 1995 by John Tsai^35^.

Renkens and Schellekens^34^, and John Tsai’s^35^ research was inconclusive because of doubt over whether it had a clear advantage over the tube refractometer. Therefore, the current research investigated whether this method creates an easier to use and more accurate refractometer than the tube refractometer.

## Results of the hollow prism using a translation stage

The angle of the prism together with the distance of travel of the translation stage is needed to calculate the distance of air replaced within a vacuum. From this, the refractive index can be calculated in the same way as the tube refractometer^20,21^.

4 $$ n = \left( {\frac{L + l}{L}} \right). $$

By substituting Eq. (3) into (4), Eq. (5) is used to calculate the refractive index

5 $$ n = \frac{{2 \times \left( {tan\theta \times Lt} \right) + l}}{{2 \times \left( {tan\theta \times Lt} \right)}}. $$

The angle of the prism was measured using a Zeiss Prismo Coordinate Measuring Machine (CMM). The accuracy of the CMM measurement is calculated from the CMM specification to be 0.5 µm^36^.

The prism intended for liquid refractive index measurements was not ideal for air measurements, and while the angle could be measured accurately, the optical flatness and parallelism of the sides of the prism could not. These measurements are necessary to determine the wavefront of the optical path of the prism while the prism is traversed across the laser beam. To determine this, measurements were taken with air inside the prism. It was assumed that the air inside and outside the prism was at the same temperature, pressure and humidity. These readings were used as a baseline measurement and were added to the final measurements made when there was a vacuum inside the prism.

The results of the prism refractometer were compared to the weather station method, where the air temperature, pressure and humidity were measured and the refractive index calculated using the modified Edlin equation^17,18^. In Table 1, the velocity of light compensation (V.O.L.) was calculated as most manufacturers of laser displacement systems use the V.O.L. in their software calculations^22–24^. The relation between the V.O.L. and the refractive index is shown in Eq. (6) and is the inverse of the refractive index *n.*

6 $$ {\text{V}}.{\text{O}}.{\text{L}}. \, = n^{{ - {1}}} . $$

**Table 1:** Results for the prism refractometer data compared to the modified Edlin equation using weather station measurement calculations.

Stage moved		Prism readings								
Transverse laser reading, *Lt* (mm)	Air length replaced, *L* (nm)	Laser reading in air (nm)	Laser reading in vacuum (nm)	Laser reading combined, *l* (nm)	Air temp (°C)	Air pres (mbar)	Air humid (%RH)	V.O.L. Edlin eq	V.O.L. prism	Diff. in V.O.L. between Edlin eq. and prism refract
Use only for Edlin calculation					20.14	866.53	45.3	0.999768124		
5	5,773,503	1305	33	1338	–	–	–	–	0.999768305	− 1.82E−07
10	11,547,006	2475	202	2677	–	–	–	–	0.999768219	− 9.50E−08
15	17,320,509	3248	770	4018	–	–	–	–	0.999768074	4.93E−08
20	23,094,012	3564	1791	5355	–	–	–	–	0.999768175	− 5.17E−08
25	28,867,514	3692	3003	6695	–	–	–	–	0.999768132	− 8.40E−09

The readings were taken in 5 mm intervals over 25 mm transverse range. As discussed previously, the first set of readings was taken while the prism was filled with air to establish the baseline measurements (see column three, Table 1). The readings were not constant where one would have expected a smaller change in the laser reading. In a flawless prism, there should be no difference in the laser reading as the prism is traversed in front of the laser beam.

The combined laser reading is recorded in column five in Table 1. The combined laser readings were used to calculate the V.O.L. in the air (see column ten, Table 1). To validate the readings, it was compared to the weather station method, and the difference in the two readings are in the last column of Table 1. The worst difference between the two methods was 1.2 × 10^–7^. The system became more accurate with a longer travel in the traverse direction. This is expected as the air/vacuum ratio is larger, and the laser readings become less sensitive to the overall accuracy.

Sets of readings were taken over a period of 2 weeks, and the standard deviation was calculated on the differences in the two methods. The worst standard deviation was 5.5 × 10^–8^ and will be discussed in detail in the next section.

The V.O.L was calculated using the Edlin equation for an Air temperature of 20.14, Air pressure of 866.53 mbar and Air humidity of 45.3%RH, to be 0.999768124.

## Added laser to optical layout

The results of the prism refractometer with the translation showed an agreement of 1.82 × 10^–7^ with the Edlin weather station method for the short travel and better than 8.4 × 10^–9^ for the longer travel of the transverse stage. This proves that this method is accurate enough to be used in dimensional metrology.

The results over a short period was very promising, but when the system was used over a longer period (a few days), it did not agree with the weather station method to the same accuracy. The reason was the drift in the zero position. Although the laser beam for the zero position was through a very small amount of vacuum compared to the laser beam through the air, it was enough to drift over time compared with the laser through the air. The drift over a few days was as much as 50 nm between the two laser beams.

**Figure 4:**
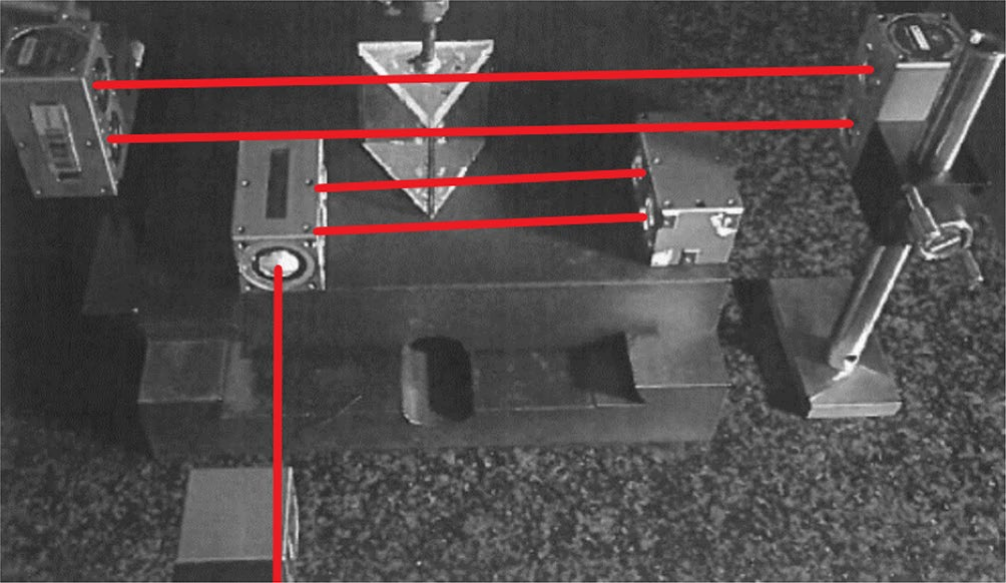
Setup with extra laser beams to continue measuring the zero position.

A new optical layout, Fig. 4, was used by adding an extra pair of laser beams to the system. This pair of laser beams was used to constantly measure the zero position. The optics of the second laser was in such a position that it will move with the translation stage and always measure the same position of the prism. This second laser reading will be used to correct the first laser reading for the zero-position drift and will substantially improve the long-term accuracy.

The new optical layout was tested with both laser beams at the zero position over a few days. The difference between the two laser readings was less than the uncertainty in the laser readings.

## Uncertainty calculations

The uncertainty for the Edlin weather station method was calculated by approximating the uncertainty in the measurement for each of the sensors. The uncertainty of the measurements was taken not only from the uncertainty of the calibration but also the accuracy of each sensor during the measurements. This was necessitated by the fact that the sensors are calibrated under near-ideal conditions but the measurements were performed in lesser conditions in the laboratory.

The uncertainty of the air temperature measurements was estimated to be 0.05 °C, which resulted in an uncertainty of 5.1 × 10^–8^ in the refractive index; the air humidity uncertainty was estimated to be 2% RH, resulting in an uncertainty of 1.7 × 10^–8^ in the refractive index; and lastly, the air pressure uncertainty was estimated to be 0.1 mbar, and this resulted in an uncertainty of 2.7 × 10^–8^ in the refractive index. When combining these uncertainties using the root sum square method, the overall uncertainties from the weather station method were 5.9 × 10^–8^ in the refractive index^21,37^.

The uncertainty for the prism refractometer was calculated using Eq. (5), where the sensitivity coefficients were calculated for each contributor. The Laser system was calibrated directly against the National Standard in South Africa, an Iodine stabilised HeNe laser. The CMM was calibrated but for the uncertainty calculations the manufacturing specification was used^36^. In Table 2, the uncertainty budget for the refractive index was only calculated for the 25 mm position, as this is the most accurate.

**Table 2:** Uncertainty budget for prism refractometer at the 25 mm position only.

Sym	Description	Value	Uncertainty	Sensitivity coefficient	Uncertainty contributor	Significance %
*q*	Angle of prism (rad)	0.52359	0,000011	− 530 × 10^–6^	− 6 × 10^–9^	2
*Lt*	Translation distance (μm)	25,000	0,5	− 9.3 × 10^–9^	− 3 × 10^–9^	1
*l*	Difference in laser reading, including repeatability (um)	6677	0,002	35 × 10^–6^	40 × 10^–9^	97
Combined uncertainty					4 × 10^–8^	
Expanded uncertainty					8.4 × 10^–8^	

Apart from the contributors in the table, the imperfections of the prism faces were calculated by measuring the baseline readings. These measurements were taken in air and added to the readings under vacuum. This eliminated this uncertainty, and only the repeatability in the readings was added to the total uncertainty.

**Figure 5:**
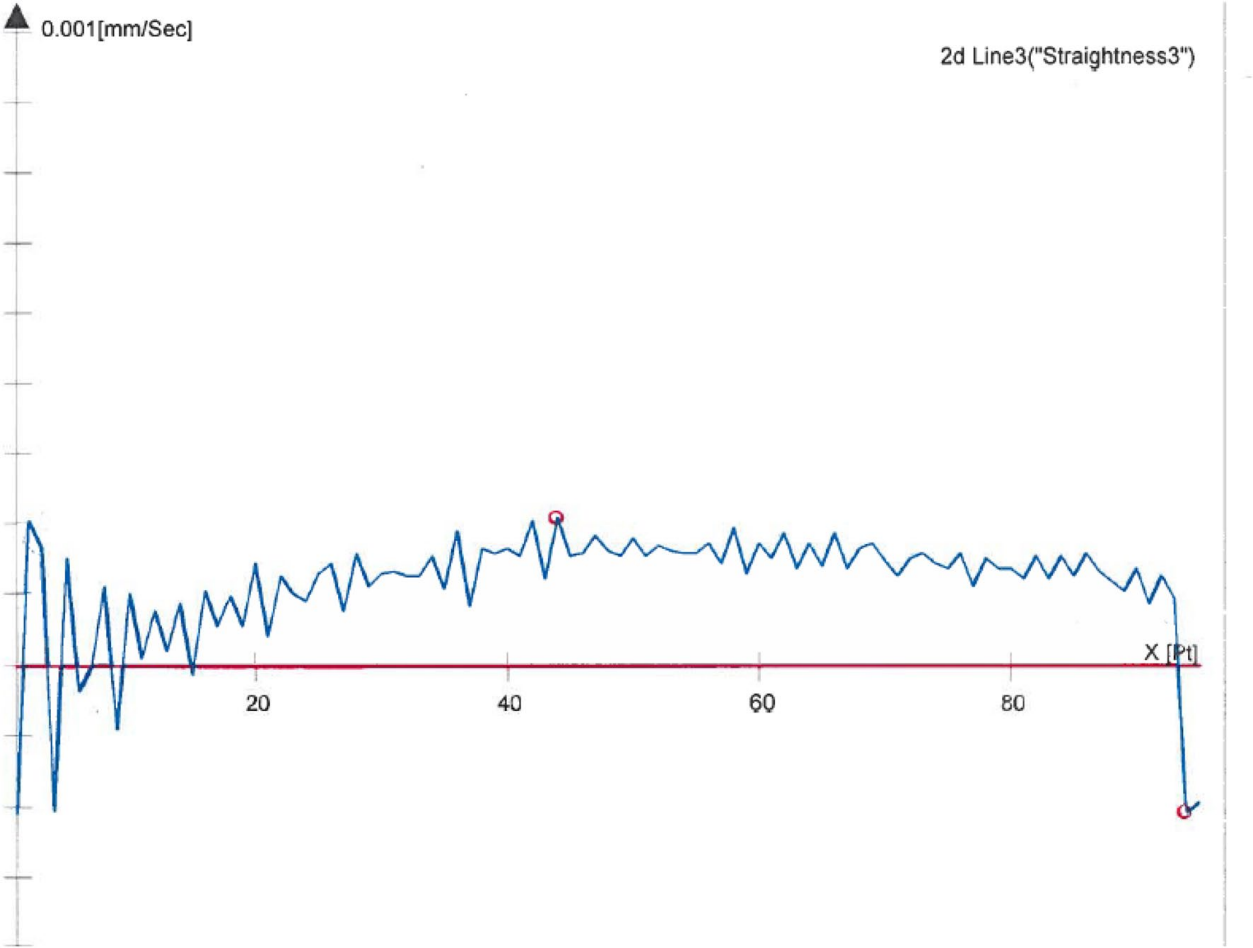
Profile of prism side under atmospheric conditions.

**Figure 6:**
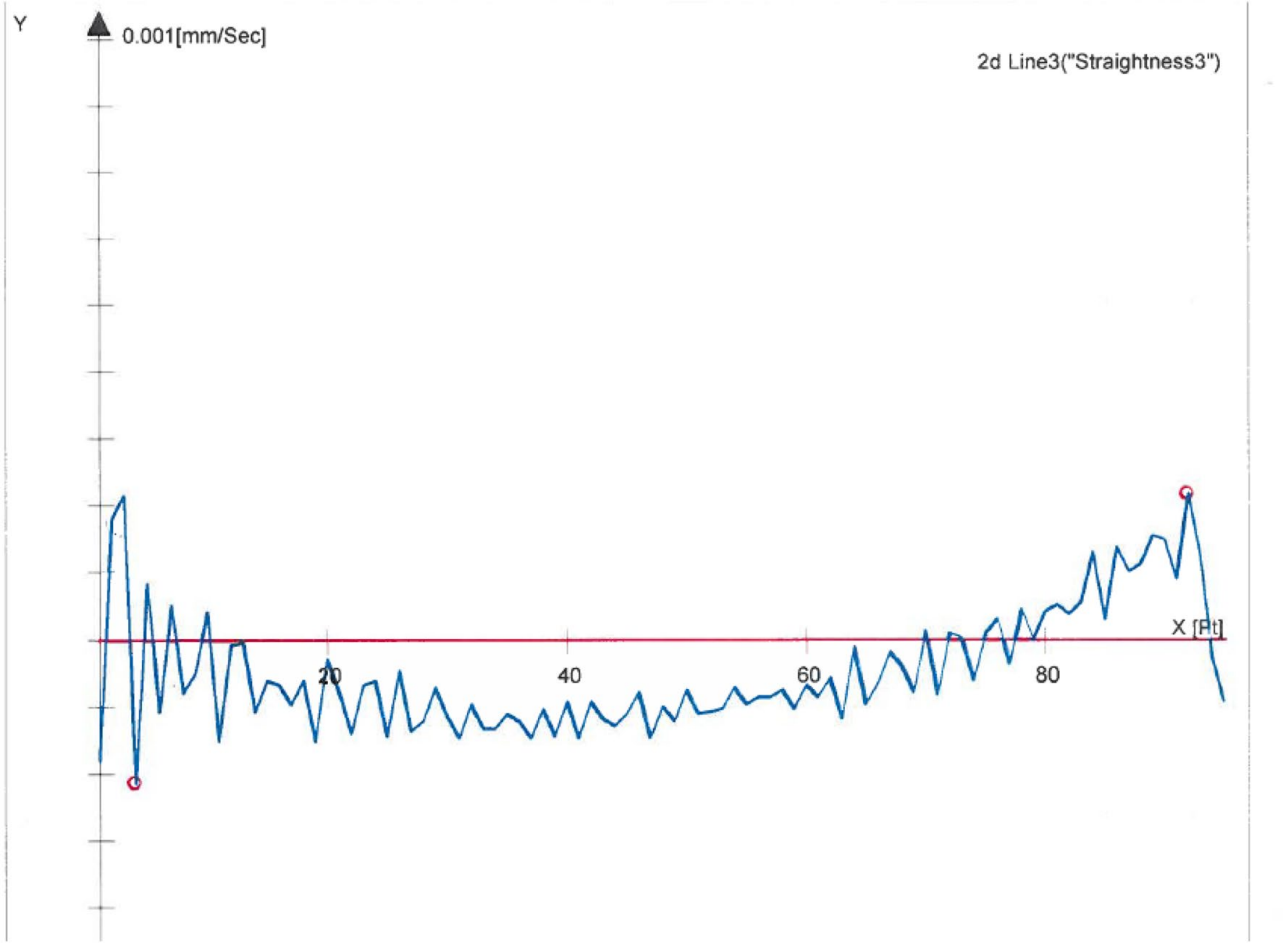
Profile of prism side under vacuum.

However, the deformation of the prism under vacuum had to be included as an uncertainty component. Straightness measurements using the Zeiss CMM were performed under normal environmental conditions with air in the prism at atmospheric conditions. The straightness of the left side of the prism is shown in Fig. 5. Thereafter, a vacuum was drawn, and the straightness of the same side was measured again and shown in Fig. 6. The straightness value between air and vacuum changed by 1 µm over the area used during the measurements, and taking the uncertainty for the deformation in the sides to be less than 0.5 µm over the area that was used during the measurements, resulted in less than 1 × 10^–9^ in the velocity of light compensation and was not included in this uncertainty budget.

The combined uncertainties using the root sum square method^37^ is 8.4 × 10^–8^ in the measurement of the refractive index.

## Conclusion

The aim of this research was to investigate the use of a permanent vacuum hollow prism as the vacuum etalon in an air refractometer in order to eliminate the use of a vacuum pump during refractive index measurements. The results of the translation showed an agreement of 1.82 × 10^–7^ with the Edlin weather station method for the short travel and better than 8.4 × 10^–9^ for the longer travel of the transverse stage.

The use of a prism as the vacuum etalon further eliminated the difficulties due to the change of the optical length of the side windows of a tube refractometer as describe by Bonsch and Potulski^20^. This is due to the optics being under the same conditions during the entire measurement. This is a great improvement in the potential accuracy of prism refractometers over tube refractometers.

Adding an extra set of laser beams to the prism refractometer improved the long-term accuracy of the refractometer, compared to other prism refractometers as describe by Renkens and Schellekens^34^, and John Tsai’s^35^. This design can now be used for a prolonged period after the prism had been translated and kept at the 25 mm position, which is a big improvement over previous designs. With these improvements, the refractometer proved advantageous not only for refractive index air measurements in dimensional metrology on a daily basis but also for high accurate research.

Future improvements to the system will focus on the commercial prism. The side windows of the prism should be manufactured of better-quality optical material and to a higher accuracy in flatness and parallelism to increase the overall accuracy of the system.

The results, both in everyday use for long-term measurements and highly accurate research, prove the original objective of this research.
